# Heat Shock Proteins: Cell Protection through Protein Triage

**DOI:** 10.1100/tsw.2010.152

**Published:** 2010-08-03

**Authors:** David Lanneau, Guillaume Wettstein, Philippe Bonniaud, Carmen Garrido

**Affiliations:** ^1^ INSERM U866, University of Burgundy, Dijon, France; ^2^ Faculty of Medicine and Pharmacy, University of Burgundy, Dijon, France; ^3^ CHU Dijon, Dijon, France

**Keywords:** heat shock proteins, proteasome, ubiquitination process, cell stress

## Abstract

Heat shock proteins (HSPs) are chaperones that catalyze the proper folding of nascent proteins and the refolding of denatured proteins. The ubiquitin-proteasome system is an error-checking system that directs improperly folded proteins for destruction. A coordinated interaction between the HSPs (renaturation) and the proteasome (degradation) must exist to assure protein quality control mechanisms. Although it still remains unknown how the decision of folding vs. degradation is taken, many pieces of evidence demonstrate that HSPs interact directly or indirectly with the proteasome, assuring quite selectively the proteasomal degradation of certain proteins under stress conditions. In this review, we will describe the different data that demonstrate a role for HSP90, HSP70, HSP27, and alpha-B-crystallin in the partitioning of proteins to either one of these pathways, referred as protein triage.

## INTRODUCTION

Heat shock proteins (HSPs) are molecular chaperones that can help cells to cope with stressful conditions. Some of them are constitutively expressed, while others are induced after different stresses. Most stresses that induce HSPs, such as heat, cytotoxic drugs, or UV irradiation, alter protein structure and cellular metabolism. The perils of protein misfolding are controlled by two mechanisms: the molecular chaperones that facilitate proper folding and the ubiquitin-proteasome system, the main pathway in eukaryotic cells that regulates the degradation of misfolded proteins.

HSPs are traditionally classified into two groups: the high-molecular-weight HSPs and the small HSP family. To the first group belong the HSP90, HSP70, and HSP60 families. They are ATP-dependent chaperones. Proteins called cochaperones bind to the HSP, stabilizing it either in an ATP- or ADP-binding conformation, thereby modulating HSP chaperone function. In contrast to the high-molecular-weight HSPs, small HSPs are ATP independent. Their functions seem to be controlled by their phosphorylation status. The most studied members of this family are HSP27 and αB-crystallin.

The ubiquitination system labels proteins for degradation by the 26S proteasome, a multisubunit protease composed of a catalytic 20S subunit and a regulatory 19S subunit. Ubiquitin is a small, highly conserved, regulatory protein that binds to proteins by covalent attachment of one or several ubiquitin monomers. The process of ubiquitination requires recognition of the target protein by the ubiquitination machinery, then activation of ubiquitin by a ubiquitin-activating enzyme (E1), its transfer to a ubiquitin-conjugating enzyme (E2) that associates with an ubiquitin ligase (E3). The E3 ligase transfers the activated ubiquitin to one or more lysine residues in the substrate and sometimes this process is facilitated by an E4 factor.

Chaperones like the HSPs and the ubiquitin-proteasome system are believed to work in a competitive manner in the selective degradation of damaged or abnormal proteins. However, a plethora of data indicates that HSPs do not have only a competitive role in the proteasomal degradation of proteins, but can also have the opposite effect and favor the degradation of selected proteins[1]. It has been shown that the main function of stress proteins is to prevent accumulation of denaturated and/or aggregated proteins, as an increase in ubiquitin-dependent degradation of proteins could prevent heat shock toxicity as efficiently as HSPs[2]. The pieces of evidence that link the proteasome system and HSPs are discussed in this mini-review. We will focus on HSP90**,** HSP70/HSC70, HSP27, and αB-crystallin.

## HSP70 AND HSP90 CHAPERONE COMPLEXES: FOLDING VS. DEGRADATION

HSC70, expressed constitutively, and HSP70, which is stress inducible, are the most studied members of the cytoplasmic 70-kDa HSP family. Their main chaperone functions are to help the folding of nascent proteins and the refolding of denatured proteins. However, most probably when the protein cannot be efficiently renatured, the chaperone, instead of trying to refold the protein, favors its degradation. The HSP70/HSC70 role in protein degradation seems rather indirect through its cochaperones CHIP and BAG-1 (Fig. 1). CHIP (C-terminus of HSP70/HSC70 interacting protein) is a ubiquitin ligase that, when interacting with HSP70/HSC70, efficiently captures unfolded proteins and ubiquitinates them, assuring the degradation of the captured protein[3,4]. CHIP possesses two functional main domains: the tetratricopeptide repeat (TPR) domain at its amino terminus, which permits its interaction with other TPR-containing proteins, such as HSP70 or HSP90, and a “U-box” domain at its carboxyl terminus that interacts with the UBCH5 family of E2 ubiquitin-conjugating enzymes[5]. This domain, which is similar to RING finger domains found, for example, in the Inhibitor of Apoptosis Proteins family, confers to CHIP the ability to ubiquitinate and to target proteins to the proteasome system[6]. CHIP participates also in the delivery of ubiquitinated proteins by interacting with the S5a component of the 19S proteasome subunit[3]. Thus, CHIP functions as a chaperone-dependent ubiquitin ligase involved in the degradation of at least some HSP70 client proteins. HSP70 acts as an adaptor for CHIP to ubiquitinate the chaperone client proteins[7,8]. CHIP can also target proteins for lysosomal degradation[9]. Two pathways of degradation called CMA (chaperone-mediated autophagy) and CASA (chaperone-assisted selective autophagy) have been reported[10]. CMA is a form of autophagy by which specific cytosolic proteins are directed for lysosomal degradation. Stress conditions, such as nutritional and cellular stresses, can activate this pathway. HSC70, associated with cochaperones, participates in CMA by recognizing a motif related to the pentapeptide KFERQ and directing it to lysomal degradation[11]. CASA is another degradation pathway involved in the maintenance of a protein assembly called Z disk that is essential for actin anchoring in striated muscle. HSC70, through the formation of a complex that includes the small HSP22 (HSPB8) and CHIP, mediates the degradation of the Z disk components[12].

Another HSP70 cochaperone involved in proteasomal degradation of abnormal proteins is BAG-1, a protein of the Bcl-2–associated athanogene (BAG)-family. BAG-1 was originally identified by its ability to associate with the antiapoptotic protein Bcl-2. BAG-1 possesses a conserved BAG domain in the region located at the C-terminus that binds the ATPase domain of HSP70[13] and acts as a nucleotide exchange factor that induces the release of substrates from HSP70[14]. BAG-1 has also a ubiquitin-like (UBL) domain at its amino terminus that allows its interaction with the 20S core and the 19S subunit of the

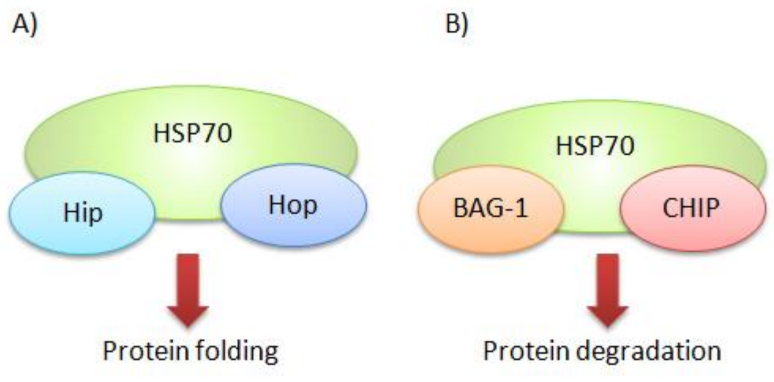

**FIGURE 1.** Folding vs. degradation. (A) The HSP70 chaperone folding complex is formed upon association with cochaperones such as Hip and Hop. Hip promotes substrate binding, while Hop promotes the recruitment of the HSP90 folding machinery. (B) The HSP70 chaperone degradation complex includes association with BAG-1 and CHIP. BAG-1 competes with Hip for binding to the ATPase domain and favors proteasome recruitment, while CHIP competes with Hop and directs the ubiquitination of chaperoned proteins.

proteasome[15].Thus, the UBL domain containing protein BAG-1 provides an additional link between chaperones and the protein degradation pathway[16]. BAG-1 and CHIP directly interact, and their coexpression increases the degradation of a model chaperone substrate[17]. Thus, these two proteins can cooperate in order to shift the activity of the chaperone systems from protein folding to degradation.

The role of HSP90 in protein degradation is often associated with HSP70, mainly because both chaperones share cochaperones through which they can transitorily associate. HSP90 assures the stability of hundreds of proteins known as HSP90 client proteins. Its broad clientele includes structurally and functionally different proteins that include a growing range of protein kinases, a variety of nuclear hormone receptors, cell surface receptors, such as Her2/neu, transcription factors, such as Hypoxia Inducible Factor (HIF)-1α, and many others[18]. The molecular basis for HSP90's specificity for client proteins is largely unknown and close homologue proteins can demonstrate a different dependence on HSP90. When the protein is not chaperoned by HSP90 any more, i.e., when this protein failed to fold properly or after treatment with HSP90 inhibitors, the client protein is ubiquitinated by an E3 ligase and degraded by the proteasome[19]. This can be done through CHIP that, although its main partner is HSP70, can also bind to the TPR domain of HSP90[20,21]. However, the mechanism of this CHIP-dependent ubiquitination is still unclear. Another possibility is that HSP90, through a transient interaction with HSP70, transfers that client protein to HSP70 and it is CHIP, associated to HSP70, that will ubiquitinate and drive the client protein to the proteasome machinery[22,23,24] (Fig. 2). In any case, CHIP, as a chaperone-dependent ubiquitin ligase, mediates quality control monitoring by targeting proteins for degradation. However, only a partial overlap exists between the proteins reported to be targeted by CHIP and the client proteins of HSP90, indicating that other proteins that do not bind HSP90 are ubiquitinated by CHIP. Conversely, other E3 ligases than CHIP must be involved in HSP90 client proteins ubiquitination-proteasomal degradation[25]. In this way, the E3 ubiquitin-protein ligase Triad3A forms a complex with Receptor Interacting Protein 1 (RIP-1) and HSP90, and mediates RIP-1 ubiquitination and proteasome degradation after HSP90 inhibition by geldanamycin[26].

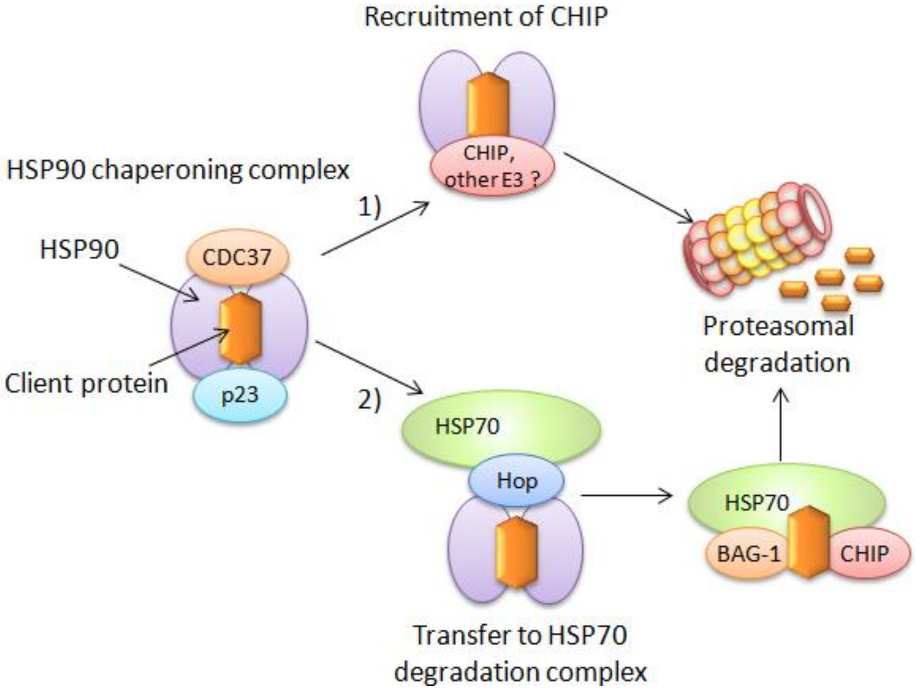

**FIGURE 2**. HSP90 client proteins degradation. HSP90 client proteins are stabilized in an HSP90 chaperone complex that includes the cochaperones p23 and CDC37. When the protein fails to fold properly or after treatment with HSP90 inhibitors, the client protein is ubiquitinated and targeted to the proteasome. This can be done either (1) by the recruitment of CHIP or another E3 ligase on the HSP90 complex or (2) by the transfer of the client protein to the HSP70 degrading complex.

## A DIRECT PARTICIPATION IN THE UBIQUITIN-PROTEASOME SYSTEM FOR HSP27 AND αB-CRYSTALLIN

HSP27 and αB-crystallin are ubiquitous proteins whose expression is induced in response to a wide variety of physiological and environmental stresses. These proteins are considered as powerful molecular chaperones whose main function is to prevent stress-induced accumulation of aggregated proteins. The dynamic organization of HSP27 and αB-crystallin oligomers appears to be a crucial factor that controls the activity of these proteins. These HSPs can form large oligomers of up to 800 kDa. The dimer seems to be the building block for these multimeric complexes. Oligomerization is a highly dynamic process that depends on the phosphorylation status of the protein. Phosphorylation induces modifications both in the oligomer size and the chaperone-like activity[27]. Dephosphorylation of HSP27 favors large oligomer formation. These large oligomers are believed to serve as “reservoirs” of unfolded or misfolded proteins. A mutant of HSP27 that mimics constitutively phosphorylated HSP27 only gives small oligomers and is quite inefficient to inhibit protein aggregation ([28] and unpublished data). Human HSP27 is phosphorylated at $Ser^{15}$, $Ser^{78}$, and $Ser^{82}$. This phosphorylation is a reversible process catalyzed mainly by the kinase MAPKAP 2/3, downstream of p38 kinase[29]. αB-crystallin is also phosphorylated at three serine residues, namely $Ser^{19}$, $Ser^{45}$, and $Ser^{59}$. In contrast to HSP27, phosphorylation *in vivo* of αB-crystallin distinct serine residues appears to be regulated independently, i.e., p44/42 MAP kinase and MAPKAP kinase-2 are responsible for $Ser^{45}$ and $Ser^{59}$ phosphorylation, respectively[30]. The kinase responsible of the phosphorylation of $Ser^{19}$ is still unknown.

HSP27 enhances the catalytic activity of the 26S proteasome machinery and increases the degradation of ubiquitinated proteins in response to stressful stimuli. In contrast to HSP70, HSP27 directly interacts with the 19S proteasome subunit and associates to ubiquitin, with higher affinity for long ubiquitin chains than for monoubiquitin[31]. Under stress conditions, HSP27 favors the ubiquitination of proteins like I-

κBα, p27$^{kip1}$, and the transcription factor GATA-1. The role of HSP27 in protein degradation demonstrates a certain degree of specificity. For example, HSP27 facilitates the degradation of proteins like I-κBα or p27$^{Kip1}$, while it does not affect that of other cell cycle proteins like cyclin A, cyclin E, cyclin D1, or β-catenin[27].

Ubiquitination utilizes a multistep enzymatic pathway in which, although the enzymatic activity required for substrate modification can be carried out by the E1, E2, and E3 enzymes alone[32], the E3-substrate interaction may be facilitated by E4 factors. The E4 factors increase ubiquitination efficiency in a substrate-specific manner, either by accelerating the transfer of ubiquitin chains from the E3 ligase to the substrate or merely by providing a scaffold. Herein, our data, therefore, raise the interesting possibility that the chaperone HSP27 might be an E4 factor, a scaffold to approach the E3 ubiquitin ligase to the target to be modified by ubiquitination.

The ubiquitin-proteasome system itself plays a major role in the modulation of apoptosis. Inhibitors of the proteasome, such as bortezomib, induce apoptosis and the expression of HSPs and, more particularly, HSP27[33], whose expression level in lymphoma cells correlated with resistance to bortezomib[34]. It is very likely that the function of HSP27 in protein triage is part of the chaperone overall protective effect. HSP27 could indirectly affect apoptosis by facilitating the degradation of death regulatory proteins. Interestingly, one of these regulatory proteins is the antiapoptotic transcription factor NF-κB, whose activation strongly depends on the ubiquitin-proteasome system that must degrade the NF-κB inhibitor (I-κBα). We have demonstrated that HSP27 stimulates the degradation of I-κBα and thereby increases the activity of NF-κB. A dominant-negative NF-κB mutant can reduce the cytoprotective action of HSP27[35], underscoring the physiological importance of the NF-κB system for apoptosis modulation by HSP27. On the other hand, stress conditions, such as staurosporine treatment, provoke a transient cell cycle arrest with an accumulation of cells in the $G_0/G_1$ phase. By favoring the ubiquitination and proteasomal degradation of p27$^{kip1}$ under those stress conditions, HSP27 avoids the p27$^{kip1}$-mediated arrest in the $G_0/G_1$ phase and facilitates the passage of cells to the S phase. We believe that this effect contributes to HSP27-mediated cytoprotection, by allowing cells to resume proliferation rapidly, once the stress has been removed[27]. Concerning GATA-1, our data suggest that HSP27 triggers its ubiquitination and degradation when acetylated. By doing so, HSP27 participates in the tight control of GATA-1 turnover necessary for erythrocytes to survive while differentiating[36].

HSP27 has also been shown to decrease, instead of favoring, the ubiquitination and proteasomal degradation of the eukaryotic transcription factor 4E (elF4E)[37]. This effect could be explained by the effect of HSP27 in the sumoylation process. Sumoylation is an ubiquitination-related process that ends up in the covalent link of a molecule of SUMO (small ubiquitin-like modifier) in a lysine residue, the same amino acids that can be modified by ubiquitin. Therefore, one possible consequence of sumoylation is to interfere with the ubiquitination process. In the sumoylation process, E3 enzymes are not strictly required[32]. They accelerate and facilitate the process. Therefore, the E3 SUMO protein would be the equivalent of an E4 protein in the ubiquitination process. We have demonstrated that HSP27 facilitates modification by SUMO of certain proteins[38]. HSP27 seems to act as an E3-like protein in the sumoylation process. Confirming this hypothesis, we and other authors have found that HSP27 is able to bind to Ubc9, the only E2 SUMO conjugating enzyme described so far[38,39]. One of the targets of HSP27 for sumoylation is the transcription factor Heat Shock Factor-1 (HSF-1). HSP27, under the form of nonphosphorylated large oligomers, induces HSF-1 sumoylation, thereby blocking the transcription factor activity[38]. A mutant of HSP27 that mimics HSP27 constitutively phosphorylated is unable to bind to Ubc9 and thereby to induce HSF-1 sumoylation. Interestingly, phosphorylated HSP27 is the form of the protein that seems involved in the modification by ubiquitination, at least of the targets analyzed so far. A challenging hypothesis will be that depending on the needs of the cell, HSP27 will control the degradation of a certain set of proteins by the modulation of its phosphorylation status. In accord with this hypothesis, HSP27, only after different stresses, has been shown to induce the degradation of proteins. Interestingly, HSP27 is rapidly phosphorylated after many different (external or physiological) stresses[29,36]. Since sumoylation can interfere with ubiquitination, we can foresee that HSP27, by modulating its phosphorylation status, may induce either ubiquitination or sumoylation, thereby controlling the fate of the protein (Fig. 3). Phosphorylation of HSP27, by inducing the disaggregation of HSP27 multimers, could also control the chaperone cellular localization and thereby its targets. Phosphorylated small HSP27 forms can be found both in the nucleus and cytosol, probably because their size allows them an easier traffic between the two cellular compartments. Therefore, HSP27 could induce the degradation of both cytosolic and nuclear proteins. In contrast, when the chaperone gets dephosphorylated and large oligomers form, they get trapped in the nucleus. Large oligomers are therefore more abundantly found in the nucleus and we can foresee that the targets of HSP27 for sumoylation must preferentially be nuclear proteins, such as transcriptions factors.

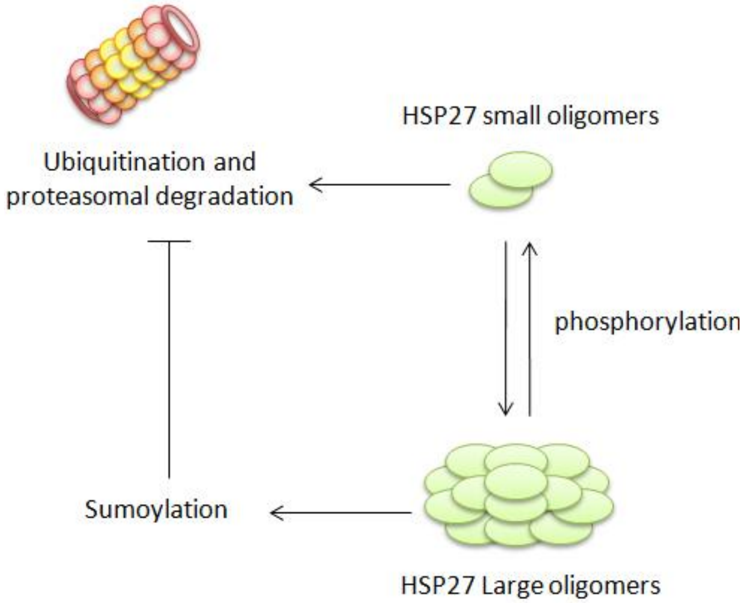

**FIGURE 3**. Hypothetic model of how HSP27 may control protein degradation. The oligomerization of HSP27 depends on its phosphorylation status. When phosphorylated, HSP27 forms small oligomers that mediate the ubiquitination and proteasomal degradation of proteins. HSP27 large oligomers are involved in the sumoylation of proteins that might "compete" with and thereby inhibit ubiquitination.

Another small stress protein that has been described to interact directly with the ubiquitin-proteasome machinery is αB-crystallin, which associates with one of the 14 subunits of the 20S proteasome, namely the C8/α7 subunit[40]. This interaction might affect the assembly of the proteasome complex or facilitate the degradation of unfolded proteins bound to αB-crystallin. When $Ser^{19}$ and $Ser^{45}$ of αB-crystallin are phosphorylated, the protein can interact with FBX4, which is a component of the ubiquitin-ligase SCF (SKP1/CUL1/F-box)[41]. αB-crystallin together with FBX4 governs ubiquitination substrate specificity. One of the substrates is cyclin D1[42,43,44]. Another target for αB-crystallin ubiquitination-proteasomal degradation is MyoD. Again this effect seems dependent on the phosphorylation of αB-crystallin[45].

## HSP PROTEASOME-DEPENDENT FUNCTIONS IN NEURODEGENERATIVE DISEASES

The clearest evidence of the implication of HSPs in protein degradation comes from neurodegenerative disorders that are characterized by a proteasomal dysfunction. A common feature of these neurodegenerative disorders, including Alzheimer's disease[46], Parkinson's disease[47], Alexander's disease[48], and Creutzfeld-Jacob syndrome[49], is the deposition of improperly folded proteins in fibers, inclusion bodies, and plaques in the nervous system. These events are associated with an increased expression of HSP27 and αB-crystallin that are typically found in association with insoluble protein aggregates and ubiquitin[50]. The ability of HSP27 to interact directly with ubiquitin could account for the described colocalization of HSP27 with ubiquitinated proteins and the 20S proteasome in cytoplasmic inclusion bodies called "aggregosomes" that characterize some of these degenerative diseases[51]. Furthermore, upon proteasome inhibition, inclusion bodies accumulate and ubiquitin-conjugated proteins appear in insoluble fraction together with chaperones HSP27, HSP70, and HSP90[52], suggesting a role for these HSPs in protein proteasomal degradation *in vivo*. *In vitro*, overexpression of HSP27 enables astrocytes to prevent irreversible damage and to recover after stress-induced proteasomal inhibition. In Alzheimer's disease, Tau, a neuronal microtubule binding protein, is hyperphosphorylated and accumulates in the neurofibrillary tangles (a hallmark of Alzheimer's disease). It has been reported that HSP27 binds to hyperphosphorylated Tau. This association favors Tau degradation and thereby a reduction in Tau levels[53,54]. HSP70 and HSP90 have also been involved in Alzheimer's disease by maintaining Tau in a soluble and functional conformation, preventing its aggregation[55]. Interestingly, HSP90 inhibition reduces the intracellular level of two phosphorylated forms of Tau involved in Alzheimer's disease.

Finally, accumulation of α-synuclein constitutes the hallmark of Parkinson's disease. Overexpression of HSP70 decreases the number of cells with inclusion bodies[56] and induces a decrease in the amount of α-synuclein protein, suggesting a role of HSP70 in the promotion of refolding and/or degradation of α-synuclein[57].

## CONCLUDING REMARKS

The ability of HSPs to promote either the renaturation or the destruction of damaged proteins under stressful conditions confers on them a key role in protein quality control and in the regulation of the protein triage. What directs the HSP decision "folding vs. degradation" remains poorly understood, but most probably depends on the type and intensity of the stress stimuli (external or physiological). HSPs and the ubiquitin-proteasome system cooperate to modulate death pathways. This cooperation may be direct, as is the case for HSP27, or may be more indirect, as is the case for HSP70 and HSP70 with its cochaperones CHIP and BAG-1. A plausible scenario emerges in which, under stress conditions, HSPs like HSP27 or HSP70 become transiently essential to assure a simpler and more rapid degradation of a selected panel of proteins, thus facilitating the survival of the cell.

Interestingly, as for all the chaperone functions of the HSPs, their role in the proteasomal degradation of unfolded substrates demonstrates a certain degree of specificity. For example, HSC70 is necessary for the degradation of actin, αB-crystallin, glyceraldehyde-3-phosphate dehydrogenase, β-lactalbumin, and histone H2A, while it is not required for the degradation of bovine serum albumin, lysozyme, and oxidized RNase A[58]. Similarly, by interacting with CHIP, HSP70 and HSP90 specifically favor the degradation of the parkin protein, CFTR (cystic fibrosis transmembrane-conductance regulator), the glucocorticoid receptor[22], and the nucleophosmin-anaplastic lymphoma kinase (NPM-ALK)[59]. HSP27 facilitates I-κBα and $p27^{Kip1}$ degradation, yet does not affect β-catenin, cyclin A, cyclin E, or cyclin D1 degradation[27]. What directs this specificity is another still-unknown question.

## ACKNOWLEDGMENTS

The CG group has the label from La Ligue Contre le Cancer. We thank the support of La Ligue Contre le Cancer, the Institut National du Cancer, and the Conseil Regional de Bourgogne. DL is the recipient of a doctoral fellowship from ARC (Association pour la Recherche sur le Cancer).
